# Coexpression of the High Molecular Weight Glutenin Subunit 1Ax1 and Puroindoline Improves Dough Mixing Properties in Durum Wheat (*Triticum turgidum* L. ssp. *durum*)

**DOI:** 10.1371/journal.pone.0050057

**Published:** 2012-11-21

**Authors:** Yin Li, Qiong Wang, Xiaoyan Li, Xin Xiao, Fusheng Sun, Cheng Wang, Wei Hu, Zhijuan Feng, Junli Chang, Mingjie Chen, Yuesheng Wang, Kexiu Li, Guangxiao Yang, Guangyuan He

**Affiliations:** China-UK HUST-Rres Genetic Engineering and Genomics Joint Laboratory, The Genetic Engineering International Cooperation Base of Chinese Ministry of Science and Technology, Chinese National Center of Plant Gene Research (Wuhan) HUST Part, The Key Laboratory of Molecular Biophysics of Chinese Ministry of Education, College of Life Science and Technology, Huazhong University of Science and Technology (HUST), Wuhan, China; TGen, United States of America

## Abstract

Wheat end-use quality mainly derives from two interrelated characteristics: the compositions of gluten proteins and grain hardness. The composition of gluten proteins determines dough rheological properties and thus confers the unique viscoelastic property on dough. One group of gluten proteins, high molecular weight glutenin subunits (HMW-GS), plays an important role in dough functional properties. On the other hand, grain hardness, which influences the milling process of flour, is controlled by *Puroindoline a* (*Pina*) and *Puroindoline b* (*Pinb*) genes. However, little is known about the combined effects of HMW-GS and PINs on dough functional properties. In this study, we crossed a *Pina*-expressing transgenic line with a *1Ax1*-expressing line of durum wheat and screened out lines coexpressing *1Ax1* and *Pina* or lines expressing either *1Ax1* or *Pina*. Dough mixing analysis of these lines demonstrated that expression of 1Ax1 improved both dough strength and over-mixing tolerance, while expression of PINA detrimentally affected the dough resistance to extension. In lines coexpressing *1Ax1* and *Pina*, faster hydration of flour during mixing was observed possibly due to the lower water absorption and damaged starch caused by PINA expression. In addition, expression of 1Ax1 appeared to compensate the detrimental effect of PINA on dough resistance to extension. Consequently, coexpression of 1Ax1 and PINA in durum wheat had combined effects on dough mixing behaviors with a better dough strength and resistance to extension than those from lines expressing either *1Ax1* or *Pina*. The results in our study suggest that simultaneous modulation of dough strength and grain hardness in durum wheat could significantly improve its breadmaking quality and may not even impair its pastamaking potential. Therefore, coexpression of 1Ax1 and PINA in durum wheat has useful implications for breeding durum wheat with dual functionality (for pasta and bread) and may improve the economic values of durum wheat.

## Introduction

Wheat is one of the “big-three” cereal crops in the world. Its success results from not only its adaptability to a wide range of climatic conditions but also its unique end-use quality which allows it to be processed into a range of flour-based food, such as bread, pasta and noodles [1]. Wheat end-use quality mainly derives from interrelated characteristics: the contents and compositions of gluten proteins and grain hardness [2]. On one hand, wheat gluten proteins predominantly determine the rheological properties of dough and thus confer the unique viscoelastic properties on dough [3]. On the other hand, grain hardness determines the milling process of flour and the physical nature of the milled products, and therefore strongly influences a bundle of quality traits [4].

Gluten proteins consist of monomeric gliadins and polymeric glutenins. In particular, the high molecular weight glutenin subunits (HMW-GS) are especially important as they are major determinants of the functional properties of wheat dough. Hexaploid wheat contains six HMW-GS genes, with tightly linked pairs of genes encoding x-type and y-type subunits located at *Glu-A1*, *Glu-B1* and *Glu-D1* loci on the chromosomes of 1A, 1B and 1D, respectively. Particularly, HMW-GS subunits 1Ax1, 1Dx5 and 1Dy10 are associated with strong dough and good breadmaking quality in bread wheat, durum wheat and Tritordeum, respectively, by transgenic technology [5], [6], [7], [8].

Grain hardness is controlled by two closely linked genes, *Pina* and *Pinb*, located at the *Hardness* locus (*Ha*) on the short arm of chromosome 5D [9], [10], [11]. PINA and PINB compose the grain hardness marker protein, friabilin, which is found in larger amounts on soft wheat starch than that on hard wheat, and absent in durum wheat [12], [13]. Recent results have demonstrated that mutations in either the *Pina-D1a* allele or the *Pinb-D1a* allele are associated with hard texture, with both wild-type *Pin* genes (the *Pina-D1a* allele and the *Pinb-D1a* allele) resulting in soft-texture phenotype [11], [14]. The causative role of *Pin* genes in kernel hardness has been fully established by transgenic lines of rice, wheat and maize expressing *Pina* and/or *Pinb* [15], [16], [17], [18], [19].

Durum wheat (*Triticum turgidum* L. ssp. *durum*) accounting for about 5% of wheat grown in the world is mainly used for pasta and couscous and also used for leavened and flat breads in Mediterranean and the Middle East countries [2]. Besides the primary importance of breeding durum wheat cultivars with superior pastamaking quality, there is increasing interest in using durum for breadmaking. However, the absence of the D-genome is considered partly responsible for the relative poor breadmaking quality of durum wheat [20]. Furthermore, the extreme hardness of durum wheat grains, due to the absence of *Pin* genes on the D-genome, increases the energy consumption during milling and starch damage levels, exerting detrimental effects on breadmaking quality of durum wheat [21]. Studies on improvement of breadmaking quality of durum wheat also suggest that it may be necessary to achieve a balance of gluten strength and extensibility with increased overall dough strength [22].

We have reported that overexpression of HMW-GS subunit 1Ax1 in several durum wheat cultivars resulted in increased dough strength [7]. More recently, we introduced wild-type *Puroindoline a* gene (the *Pina-D1a* allele) into a durum wheat cultivar Luna [23]. In this study, we used conventional crossing of these transgenic lines to develop lines combining both the *1Ax1* and *Pina* transgenes which determines dough strength and grain hardness, respectively. These transgenic isolines that expressed transgenic *1Ax1*, *Pina*, or *1Ax1* and *Pina* allow us to study the separate and combined effects of 1Ax1 and PINA on grain hardness and dough mixing properties, and to gain new insight into influences of combinations of dough strength and grain hardness on the end-use quality of wheat.

## Materials and Methods

### Plant Materials

Both transgenic line expressing *Pina* (the *Pina-D1a* allele) and *1Ax1*, respectively, were produced by transformation of durum wheat cv. Luna, which expresses only HMW-GS pair 1Bx7+1By8 [7], [23]. *Pina*-expressing line was generated by particle bombardment with the plasmid pUbi-pinA, which contains the *Pina-D1a* gene driven by the constitutive maize ubiquitin promoter, in combination with the plasmid pCa-neo, which contains the neomycine phosphotransferase II (*nptII*) gene under the control of cauliflower mosaic virus 35S (CaMV 35S) promoter. *1Ax1*-expressing line was transformed with the plasmid p1Ax1 [24] and pAHC25 [25]. The plasmid pAHC25 contains the *bar* gene, conferring the resistance to the herbicide BASTA, and the *uidA* gene, encoding for β-glucuronidase (GUS), both under the control of the constitutive maize ubiquitin promoter. The plasmid p1Ax1 contains a 7.0 kb *Eco*RI genomic fragment including the coding sequence of the *Glu-A1-1a* (1Ax1) gene flanked by 2.2 kb and 2.1 kb of 5′ and 3′ sequence, respectively.

Crosses of the two transgenic lines were carried out in 2007. F_1_ plants were screened by PCR amplification of *Pina* gene as described by Luo et al. [23], and F_2_ seeds harvested in growth chamber were analyzed by sodium dodecyl sulfate polyacrylamide gel electrophoresis (SDS-PAGE) in order to screen cross progenies containing the transgenic *1Ax1*, *Pina* or *1Ax1*+*Pina*, respectively. Selection of homozygous progeny with stable expression of 1Ax1, PINA or 1Ax1+PINA was carried out in the following three years (2008 and 2010) by SDS-PAGE analysis of total storage proteins and Triton X-114 soluble proteins (see below). In addition, the presence of *bar*, *uidA* and *nptII* genes were determined by PCR in the F_3_, F_4_ and F_5_ generations (see below). We selected two homozygous F_5_ lines coexpressing *1Ax1* and *Pina* (named HP-19 and HP-245), two lines expressing transgenic *1Ax1* (named H-182 and H-293), two lines expressing transgenic *Pina* (named P-121 and P-149) and one null segregant line named N-1. Durum wheat cv. Luna was used as non-transformed control line in this study. These homozygous lines and non-transformed control were then self-pollinated and analyzed in 2011 under field conditions.

### DNA Extraction and PCR Amplification

DNA was extracted from leaves by the CTAB method. PCR screening of *Pina* was as described by Luo et al. [23]. Determination for the presence of marker genes (*bar*, *uidA* and *nptII*) were carried out using primers specific for them (Table S1).

### Field Trials

Field trials were performed in the experimental field of the Chinese National Center of Plant Gene Research (Wuhan) HUST Part (Wuhan, Hubei Province, China), under irrigation, using a randomized complete block design with two replicates. Each plot consisted of four rows, 2.5-m long, with 50 seeds per row. The space between rows was 30 cm, and the separation between plots was 50 cm. 1,000-seed weight was determined on 500 seeds from each plot per line with three replicates each (six measurements). Test weight expressed in grams per liter was determined by weighing 100 ml of seeds with three replicates per line and per plot (six measurements).

### Seed Storage Protein Characterization

For determining grain protein contents (GPC) and flour protein contents (FPC) of each line, seeds harvested from two plots were blended together. GPC and FPC were measured on grains and flours, respectively, by near-infrared reflectance spectroscopy (NIRS) method using an Infratec TM1241 Grain Analyzer (Foss North America, Silver Spring, MD) and adjusted to a 14% moisture basis (standard methods of the International Association for Cereal Science and Technology, ICC, no. 159 and no. 202).

Total storage proteins from endosperm were extracted from single kernels of transgenic and control lines according to Liu et al. [26].

To characterize storage proteins from each line, gliadins, glutenins and other proteins were sequentially extracted from 100 mg flour from each sample according to DuPont et al. [27]. The albumin/globulin, gliadin, and glutenin fractions were sequentially extracted from the same flour sample with 0.3 M NaI in 7.5% 1-propanol followed by 2% SDS, 25 mM DTT in 25 mM TRIS, pH 8.0, and precipitation of the solubilized proteins with ammonium acetate/methanol followed by acetone. The gliadin, glutenin and albumin/globulin fractions from 15 flour samples per line were separated by SDS-PAGE as described previously [26] and quantified by densitometry method using a Bio-Rad Quantity One 1-D software version 4.6.2 (Bio-Rad, Hercules, CA). Densitometry method was selected because its higher reproducibility in characterization of storage proteins than that of HPLC [28].

### Grain Hardness Measurement

Grain hardness, kernel weight and kernel diameter were measured using the Perten Single Kernel Characterization System (SKCS) 4100 (Perten Instrument North American Inc., Springfield, IL, USA) on samples of 300 seeds harvested from each plot in 2011 according to the AACC approved method 55-31 [29].

### Scanning Electron Microscopy (SEM)

Dry mature wheat kernels were cut into halves with stainless steel uncoated single-edge industrial blades at room temperature and placed on aluminum specimen stub by double-sided tape. The kernel sections were gold coated at 10 mA for 6 min to get an approximately 15–20 nm thick coating (Technics Hummer V Sputter Coater, Technics, San Jose, California, USA).Then wheat kernel sections were examined using a Hitachi SEM-600 (Hitachi High-Technologies Corp., Tokyo, Japan) at 1.5 K magnification (15 kV).

### Extraction of Triton X-114 Soluble Protein, Friabilin and Western Blotting

In order to detect the total puroindoline, Triton X-114 soluble protein was extracted from single crushed kernel using Triton X-114 (TX-114) detergent according to Giroux and Morris [14]. The TX-114 soluble proteins were electrophoretically separated by SDS-PAGE using standard method with 15% separating gels and stained overnight with Commassie blue R-250.

To determine the amounts of starch bound puroindoline, starch granule surface proteins were isolated from 100 mg of wholemeal flour as described previously [11], separated by SDS-PAGE using 13.5% T, 2.6% C gels and stained with Commassie blue R-250. For the SDS-PAGE and Western blotting analysis (see below) of total and starch-bound PINA, proteins extracted from bread wheat cv. Chinese Spring (CS, contains the *Pina-D1a* allele) and durum wheat cv. Luna (lacks *Pina* gene) were used as positive and negative controls, respectively.

To identify the total and starch-bound PINA and estimate their amounts, TX-114 soluble proteins and starch granule surface proteins, both of which were extracted from 100 mg of flour per line, were used in Western blotting using the same loading volume for each sample. Western blotting was performed using a rabbit anti-PINA polyclonal antibody raised from the recombinant PINA proteins expressed in *E. coli*. The heterologous expression of PINA protein and its purification were reported previously by Miao et al. [30]. After protein electrophoresis, gels were blotted onto nitrocellulose membranes. Membranes were blocked overnight at 4°C in 1× TBST (Tris buffered saline plus 0.1% Tween-20) containing 5% NFDM (non-fat dry milk). Anti-PINA polyclonal antibody was used at a dilution of 1∶10000 in TBST/5% BSA and incubated for 2.5 h at room temperature. Membranes were then washed with TBST for five times and incubated with 1∶4000 dilution of alkaline phosphatase conjugated goat anti-rabbit secondary antibody for 1 h at room temperature, then detected according to the manufacturer instructions. The amounts of total and starch-bound PINA were determined by densitometry analysis of Western blotting results in three biological replications using a Bio-Rad Quantity One 1-D software version 4.6.2 (Bio-Rad, Hercules, CA).

### Mixograph

Seeds from six transgenic lines, one null segregant line and one non-transformed control line harvested in 2011 were used for analysis of dough mixing properties. Prior to milling, kernel moisture was adjusted to 16% by incubation overnight at room temperature. One hundred grams of seeds per line were milled to flour with a Brabender Quandrumat Junior Mill (C. W. Brabender Instruments, Inc., South Hackensack, NJ) following AACC method 26–50 [29]. Samples were mixed to optimum water absorption and the dough mixing properties for each line were determined using a 10 g Mixograph (National Manufacturing Co., Lincoln NE) with two replicates according to the approved AACC method 54-40A [29].

Mixograph parameters were obtained from Mixsmart software version 3.8 (AEW Consulting, Lincoln, NE, commercially available through National Manufacturing Division of TMCO, Lincoln NE, USA). They include four parameters describing the heights of Mixogram curve (midline left value, MLV; midline peak value, MPV; midline right value, MRV and midline value at 8 min, MTxV) and four describing the widths of curve (midline left width, MLW; midline peak width, MPW; midline right width, MRW; and midline width at 8 min, MTxW). Other parameters were midline peak time (MPT) and the area under midline for 8 min (MTxI). Weakening slope (WS) expressing dough weakening was computed by the difference of MPV and MTxV. These mixing parameters were used for they maintain a good representation of dough properties with a minimum of information redundancy [31].

### Statistics Analysis

Data were analysed using the SPSS version 11.0 statistical software package (SPSS Inc., Chicago, Illinois, USA). The general analysis of variance and the least significant difference pairwise comparisons of means were used to determine significant difference. The statistical significance for mixing parameters from lines expressing *1Ax1* and/or *Pina* was determined using Student’s *t* test.

### Ethics Statement

The described filed studies were approved according to the document ‘The Biosafety Permit of Transgenic Plant Research: The Permit for Field Trial of Transgenic Wheat (No. 033)’, authorized by the Ministry of Agriculture of the People’s Republic of China.

## Results

We have previously reported lines of durum wheat expressing transgenic *1Ax1* with increased dough strength [7] and transgenic lines with expression of PINA [23], both of which were generated in the same durum wheat cv. Luna. To study the combined effects of 1Ax1 and PINA on dough mixing properties, these lines were used as parents to obtain hybrid lines expressing the combination of transgenic *1Ax1* and *Pina* by conventional crossing. It is the first report on the characterization of transgenic lines coexpressing *1Ax1* and *Pina* in durum wheat and on studying the combined impacts of 1Ax1 and PINA on dough mixing properties. After selection for three consecutive years, two transgenic lines coexpressing *1Ax1* and *Pina* (containing *bar*, *uidA* and *nptII* genes) were designated HP-19 and HP-245; two lines expressing *1Ax1* (containing *bar* and *uidA* genes) were designated H-182 and H-293; two lines expressing only *Pina* (containing *nptII* gene) were designated P-121 and P-149; one null segregant line selected from crossing progeny with the absence of both target genes and marker genes were designated N-1 (Figure S1). These lines, together with a non-transformed control (cv. Luna), were used to determine the separate and combined effects of 1Ax1 and PINA on the dough mixing properties.

### Analysis of Grain Hardness

**Figure 1:**
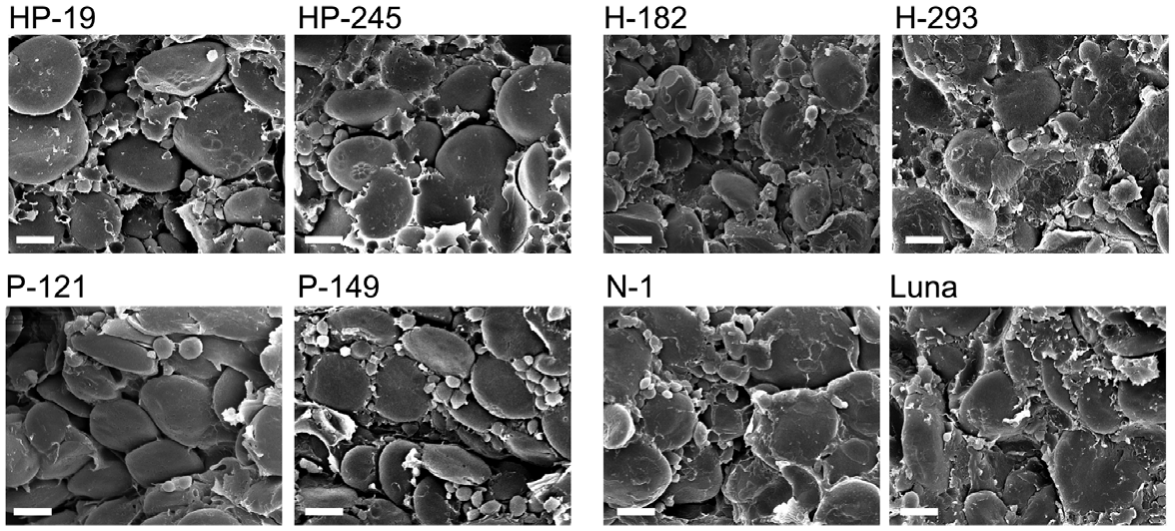
Scanning electron microscopy analyses of endosperms from transgenic and control lines. Seeds from two lines expressing *1Ax1* and *Pina* (lines HP-19, HP-245), two lines expressing *1Ax1* (lines H-182, H-293), two lines expressing *Pina* (lines P-121, P-149), one null segregant line (lines N-1) and non-transformed control cv. Luna were subjected to scanning electron microscope analysis to reveal the structure differences of endosperm. Scale bar = 10 µm.

As *Pina* was demonstrated to be a major determinant gene for grain hardness in bread wheat [18], we therefore analyzed the grain hardness of these transgenic lines, as well as kernel weight and kernel diameter, by SKCS (Table 1). Grain hardness was significantly decreased in *Pina*-expressing lines, including lines coexpressing *1Ax1* and *Pina*; while *1Ax1*-expressing lines showed similar grain hardness values with control lines. Grain hardness values of *Pina*-expressing lines were decreased to about 50, whereas lines without PINA expression had values of ca. 75. Analysis of variance (ANOVA) results did not find any significant difference in kernel weights and kernel diameters among these lines (Table 1). To further confirm the differences in kernel hardness among these lines, the endosperm structures of transgenic and control lines were imaged by using SEM and are shown in Figure 1. Endosperm structures of soft and hard wheat differ by the amount of protein matrix adhering to starch granule surface [16]. The starch granules in soft wheat appears to be round and smooth due to their weak bonding with protein matrix, while more adhesions of protein to the starch granules are observed in hard wheat [32]. As shown in Figure 1, all *Pina*-expressing lines (HP-19, HP-245, P-121 and P-149) had smoother starch granules and less adhering proteins than lines without expression of PINA, where layers of protein matrix were observed to be covered on the surfaces of starch granules. Therefore, differences in endosperm structures among these lines are in accord with their grain hardness, demonstrating that expression of PINA in durum wheat decrease grain hardness.

**Table 1:** Kernel characteristics, protein contents and Mixograph parameters of the transgenic and control lines.

Parameters	Line							
	HP-19	HP-245	H-182	H-293	P-121	P-149	N-1	Luna
Transgene	*1Ax1*+*Pina-D1a*	*1Ax1*+*Pina-D1a*	*1Ax1*	*1Ax1*	*Pina-D1a*	*Pina-D1a*	None	None
*Kernel characteristics*								
Grain hardness	52.2 c	50.8 c	76.7 a	76.9 a	51.6 c	46.0 d	73.6 b	75.3 ab
Kernel weight (mg)	31.38	33.22	34.95	35.46	34.52	36.78	35.1	37.1
Kernel diameter (mm)	1.89	1.91	2.09	2.12	1.99	2.06	2.06	2.33
1,000-seed weight (g)	30.2 g	31.7 e	32.1 d	32.7 c	33.3 b	34.3 a	32.8 c	31.1 f
Test weight (g/l)	709 cd	727 b	713 c	729 b	738 a	738 a	699 e	705 d
*Protein content*								
Grain protein content (%)	15.5 b	16.2 a	13.9 cd	13.8 d	13.2 e	14.0 cd	13.6 de	14.3 c
Flour protein content (%)	13.8 b	14.4 a	12.5 c	12.4 cd	11.7 e	12.1d	11.4 e	12.2 cd
*Mixograph*								
Midline left value (% Torque)	37.25 ab	38.49 a	35.48 ab	34.94 b	29.89 c	29.72 c	27.26 cd	25.81 d
Midline left width (% Torque)	18.92 bcd	21.03 ab	20.05 bc	17.17 bcd	15.27 cd	14.27 d	24.34 a	24.86 a
Midline peak value (% Torque)	38.11 ab	40.37 a	36 bc	35.22 cd	30.23 e	30.17 e	33.15 d	33.29 d
Midline peak width (% Torque)	20.28 c	19.56 cd	16.14 e	17.47 de	13.61 f	11.49 f	25.4 b	29.85 a
Midline peak time (min)	5.14 bc	4.5 cd	6.03 ab	6.25 a	4.37 cd	4.12 d	2.19 e	2.04 e
Midline right value (% Torque)	36.94 ab	38.77 a	34.92 bc	34.13 c	29.56 de	28.94 e	30.72 de	31.14 d
Midline right width (% Torque)	16.69 ab	18.93 a	15.67 abc	13.85 bc	12.18 c	12.21 c	15.92 abc	18.96 a
Midline value at 8 min (% Torque)	36.6 ab	37.59 a	34.98 bc	34.38 c	28.13 de	27.81 e	28.97 de	29.54 d
Midline width at 8 min (% Torque)	15.46 ab	16.52 a	16.54 a	14.91 abc	11.36 cd	9.96 d	13.65 abcd	12.43 bcd
Midline integral at 8 min (% Torque* min)	275.79 b	289.43 a	258.17 c	252.42 c	228.33 d	228.44 d	229.31 d	236.17 d
Weakening slope (% Torque)	1.51 cde	2.77 bc	1.02 de	0.84 e	2.11 cde	2.36 cd	4.17 a	3.76 ab

Values within the same parameter followed by the same letter are not significantly different at 0.05 probability level.

Protein contents determined by near-infrared reflectance spectroscopy (NIRS) method were adjusted to a 14% moisture basis.

The 1,000-seed weight varied from 30.2 g for line HP-19 to 34.3 g for line P-149; while the test weight ranged from a maximum of 738 g/l for line P-149 to a minimum of 699 g/l for the null segregant line. Although significant difference was detected in 1,000-seed weight and test weight among lines, no clear tendency was found in these traits.

### Analysis of Total and Starch-bound PINA

**Figure 2:**
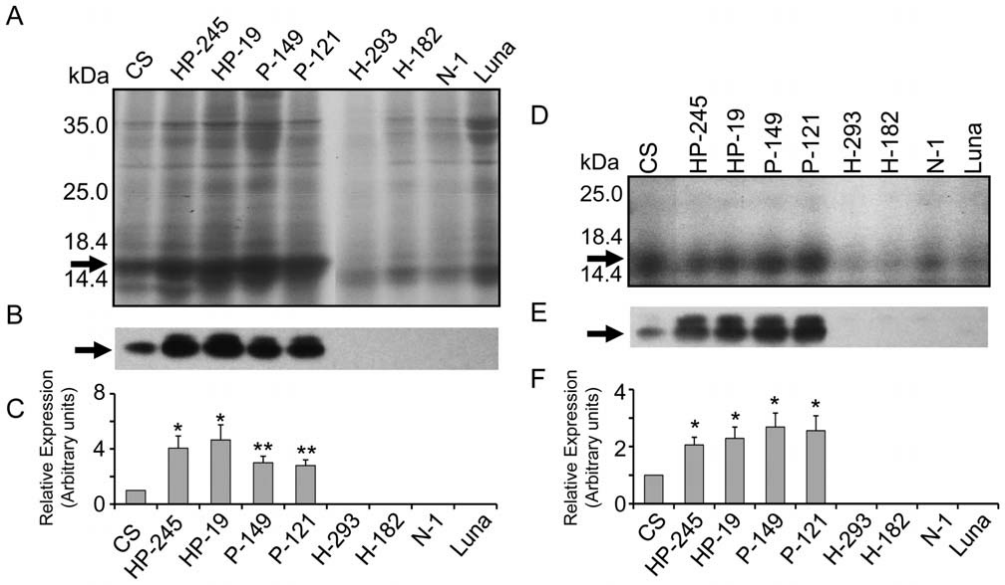
SDS-PAGE and Western blotting analyses of total and starch-bound PINA in transgenic and control lines. A. SDS-PAGE of TX-114-soluble proteins isolated from flours of transgenic and control lines. B. Western blotting results of total PINA. C. Densitometry quantification of western blotting results of total PINA. D. SDS-PAGE of starch bound puroindolines isolated from flours of transgenic and control lines. E. Western blotting results of starch-bound PINA. F. Densitometry quantification of western blotting results of starch bound PINA. PINA protein is indicated by arrow on both stained SDS-PAGE and Western blots. Data are given as mean ± SEM. *and ** indicates the significant differences with the PINA levels of control variety Chinese Spring at 0.05 or 0.01 probability level, respectively.

To investigate the expression levels of the *Pina-D1a* transgene among transgenic and control lines, total TX-114-soluble proteins were extracted and fractionated by SDS-PAGE, followed by Western blotting using a polyclonal antibody raised against the PINA peptide (Figure 2A, B and C). Soft bread wheat variety Chinese Spring (CS) has functional PINA and PINB proteins (encoded by the *Pina-D1a* and *Pinb-D1a* alleles), yielding a visible band approximately 15 kDa. Non-transformed control Luna does not express both PINs due to the lacking of the D-genome and therefore no bands corresponding to PINs was observed (Figure 2A). Four *Pina*-expressing lines showed clearly increase in PINA levels compared to that of CS, while PINA was not detected in Triton X-114 extracts from lines H-182, H-292, N-1 and Luna. Similar results were given by Western blotting analysis, with expression levels of PINA from lines HP-19, HP-245, P-121 and P-149 increasing from about 2-fold to 4-fold compared with that from CS (Figure 2C).

It has been demonstrated that the amount of PINs bound on the surface of water-washed starch granules is well associated with grain hardness and can be used to measure the proportion of PINs that are present in functional form [18]. Thus, the starch-bound PINA levels in the seeds of transgenic and control lines were investigated. Increased starch-bound PINA levels were observed, with all four *Pina*-expressing lines showing a 2- to 3-fold of starch-bound PINA levels in comparison with that of CS (Figure 2D, E and F). Briefly, similar amounts of starch-bound PINA between transgenic lines were not totally consistent with the Triton X-114 results (Figure 2), suggesting that it appears to be a limitation on the binding of PINA to starch granules.

Unexpectedly, an additional band about 18 kDa was detected in the Western blotting results of starch-bound PINA. In addition, a faint band with similar relative molecular weight was observed in the Western blotting results of total PINA. Specific detection of PINA in *Pina*-expressing lines and positive control CS but not in negative control Luna demonstrated the specificity of this polyclonal antibody. The co-presence of PINA and the 18-kDa band implied that the additional band was specifically detected by the PINA antibody. Moreover, due to the presence of reducing agents (SDS and dithiothreitol) and the heating process before loading protein samples, we eliminate the possibility of heterodimerization between PINA and some unknown proteins. For all the above reasons, we speculate that this additional 18-kDa band may be attributed to posttranslational modification of PINA protein.

### Storage Protein Characterization

The grain protein contents and flour protein contents of transgenic and control lines were compared and are shown in Table 1. The grain protein contents ranged from 13.2% to 16.2% for line P-121 and line HP-245, while the flour protein contents varied from 11.4% to 14.4% for the null segregant line and line HP-245. All transgenic lines expressing *1Ax1* had higher flour protein contents than the lines without transgenic *1Ax1* but only the protein contents of line HP-19 and HP-245 were significant higher than the others (Table 1).

**Figure 3:**
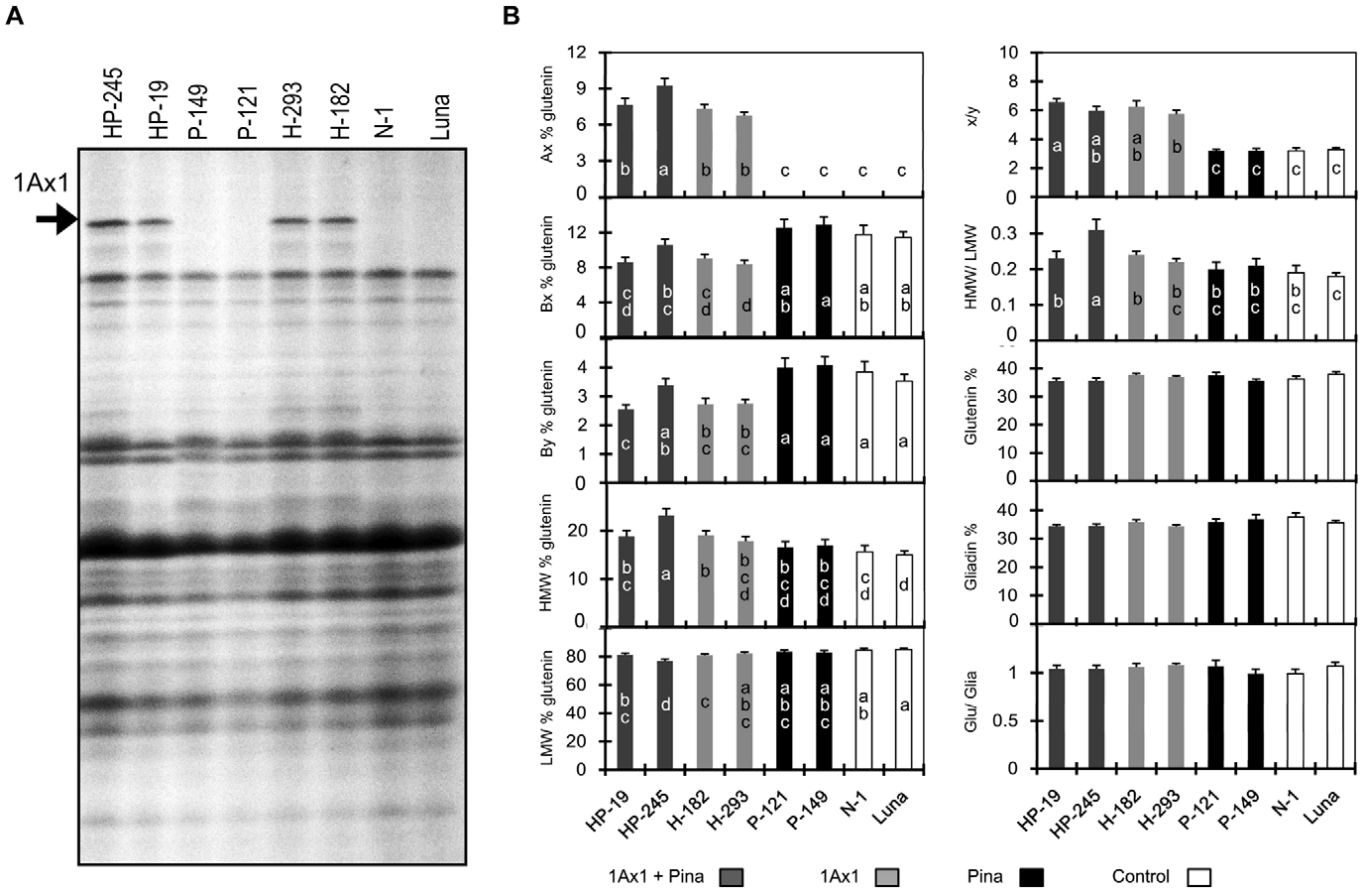
Characterization of storage proteins in transgenic and control lines. A. SDS-PAGE of seed protein extracts from transgenic lines, null segregant line and non-transformed control line. Transgenic 1Ax1 is indicated by arrow on the left side of the gel. B. Characterization of storage proteins from the transgenic and control lines. HMW % glutenin and LMW % glutenin means quantities of HMW-GS and LMW-GS, respectively, expressed related to total quantity of the glutenins (and the same for Ax, Bx and By). x/y: ratio of the x- and y- type HMW-GS. HMW/LMW: ratio of the high and low molecular weight glutenin subunits. Glutenin %: quantity of the glutenins expressed related to total proteins extracted by the sequential extraction methods (and the same for Gliadin %) [27]. Glu/Glia: ratio of the glutenins and gliadins. 1Ax1+Pina = transgenic lines coexpressing *1Ax1* and *Pina* genes (dark grey); 1Ax1 = transgenic lines expressing only *1Ax1* (light grey); Pina = transgenic lines expressing only *Pina* (black). Control = both null segregant and non-transformed control lines (white). Data are given as mean ± SEM. Values within the same characteristics of storage proteins followed by the same letter are not significantly different (*P*<0.05).

Further, SDS-PAGE results of total storage protein extracts from transgenic and control lines showed that expression patterns of major storage proteins did not appear to be affected by neither *1Ax1* gene nor *Pina* gene (Figure 3A). Moreover, to compare the protein compositions of flour samples from these lines we separated, recovered and quantified glutenins and gliadins according to DuPont et al. [27]. As shown in Figure 3B, expression of PINA did not influence the amounts and proportions of storage proteins. In contrast, the transgenic subunit 1Ax1, accounting for approximately 8% of total glutenins in four *1Ax1*-expressing lines, resulted in significant changes in the proportions of endogenous storage proteins. The increase of 1Ax1 was significantly associated with decrease in endogenous HMW-GS in three *1Ax1*-expressing lines, except for line HP-245 (Figure 3B). The endogenous Bx subunit decreased from ca. 12% in lines without 1Ax1 to ca. 8% in *1Ax1*-expressing lines, while By subunit decreased from ca. 4% to ca. 2.5%. These changes in the amounts of transgenic and endogenous HMW-GS increased the ratio of x-type/y-type HMW-GS in transgenic lines expressing *1Ax1*. Therefore, the ratios of x-type/y-type HMW-GS in the *1Ax1*-expressing transgenic lines were increased to about 6 as a consequence of the addition of foreign *1Ax1* gene. Although the expression of transgenic *1Ax1* was compensated by endogenous HMW-GS, the amounts of total HMW-GS were still increased, albeit nonsignificantly. Furthermore, increase of total HMW-GS also appeared to be compensated by LMW-GS, with the amounts of LMW-GS in four *1Ax1*-expressing lines being slightly lower than those in the other lines (Figure 3B). Particularly, the increase of HMW-GS and compensation of LMW-GS were significant in line HP-245. Finally, changes in the proportions of glutenins did not affect the amounts of total glutenins and gliadins. No significant differences in the ratio of glutenins and gliadins were observed in all cases.

### Dough Mixing Properties

**Figure 4:**
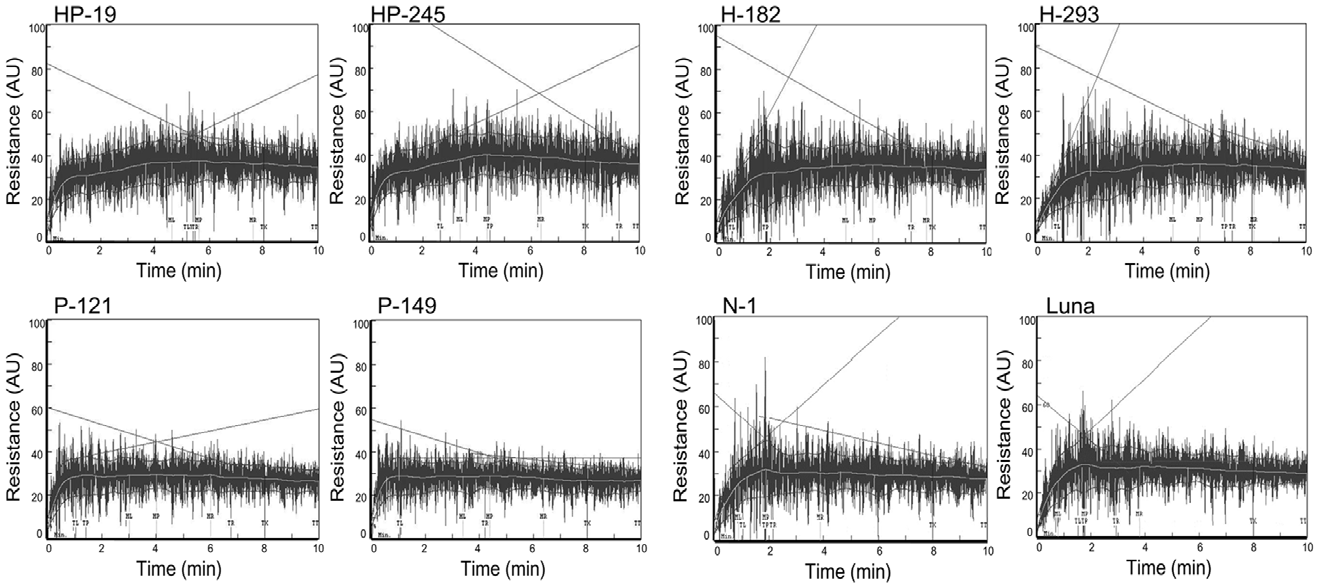
Mixograph curves of dough prepared from transgenic and control lines. Flour samples from two lines coexpressing *1Ax1* and *Pina* (lines HP-19, HP-245), two lines expressing *1Ax1* (lines H-182, H-293), two lines expressing *Pina* (lines P-121, P-149), one null segregant line (lines N-1) and non-transformed control cv. Luna were subjected to Mixograph analysis to reveal differences in dough mixing properties.

**Figure 5:**
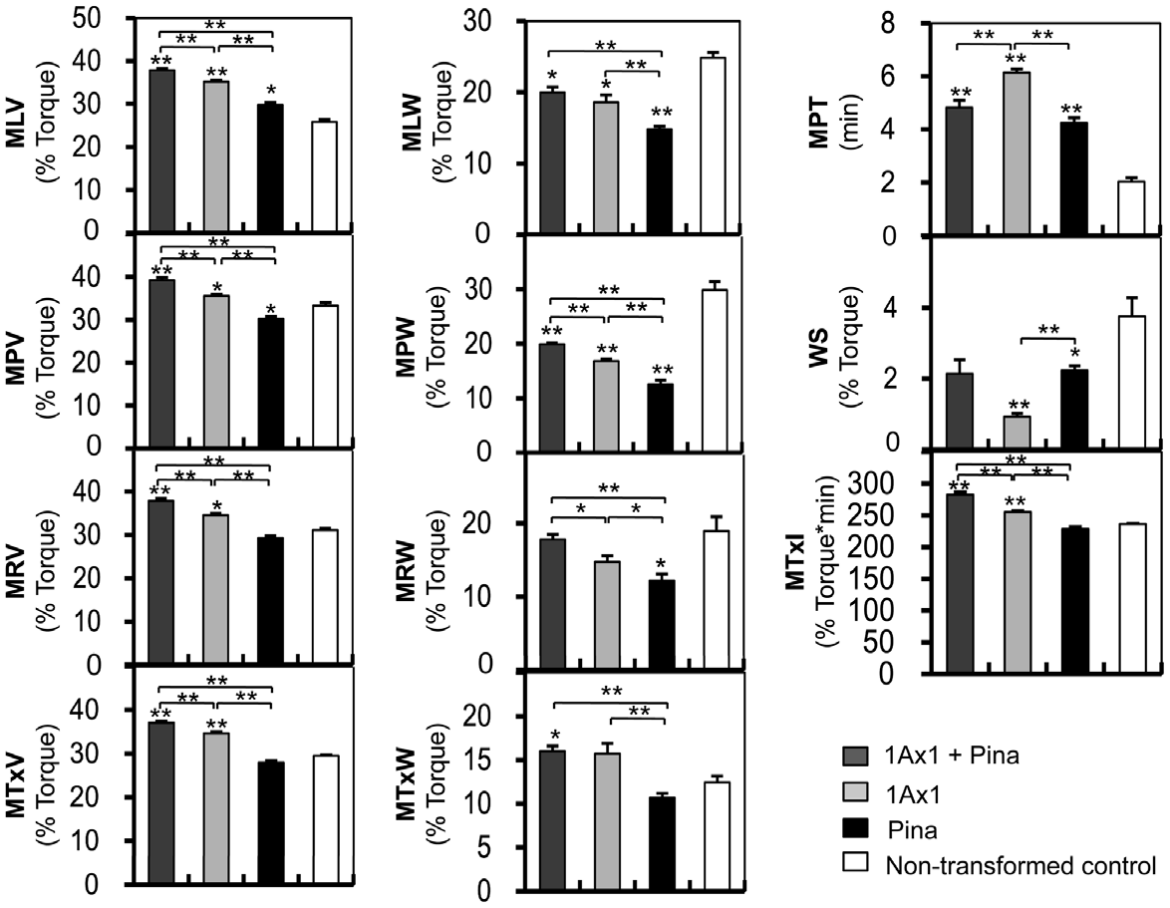
Combined effects of 1Ax1 and PINA on dough mixing properties. Eleven mixing parameters were compared by Student’s *t* test between lines coexpressing 1Ax1and Pina (lines HP-19 and HP-245, represented by dark grey bar), lines expressing only *1Ax1* (lines H-182 and H-293, represented by light grey bar), lines expressing only *Pina* (lines P-121 and P-149, represented by black bar) and non-transformed control line (cv. Luna, represented by white bar). Data are given as mean ± SEM. *and ** indicates the significant differences with mixing parameters of non-transformed control Luna at 0.05 or 0.01 probability level, respectively.

The rheological properties of transgenic and control lines were determined using a 10 g Mixograph (Figure 4). The Mixograph is widely used in cereal research as it measures a variety of rheological parameters that relate to the dough behavior in breadmaking and other food processing systems [31]. Briefly, Mixograms of lines expressing *1Ax1* were higher and wider than lines without transgenic *1Ax1* (including both *Pina*-expressing and control lines). Lines expressing *Pina* showed narrower Mixogram curves and different shapes of curve was observed in the first two minutes of mixing in comparison with those of *1Ax1*-expressing and control lines, indicating that the hydration and blended stages of dough mixing might be affected by expression of PINA [33]. We further compared the mixing parameters between lines expressing *1Ax1*, *Pina* and *1Ax1*+*Pina* (Figure 5). No significant differences were found in the mixing parameters between null segregant line (N-1) and non-transformed control (cv. Luna), suggesting that genetic transformation of wheat does not affect dough mixing properties (Figure S2). Transgenic lines expressing only *1Ax1* (H-182 and H-293) showed significant increases in parameters relating to the heights of curves (MLV, MPV, MRV and MTxV) in comparison with the non-transformed control (Figure 5). The heights of the curve after peak resistance remained stable at about 35% torque in lines H-182 and H-293, whereas those in the Luna control decreased to 29% torque. Moreover, *1Ax1*-expressing lines showed significant decreases in MLW and MPW, while a non-significant increase of MTxW was observed in lines H-182 and H-293. MPT and MTxI values for lines H-182 and H-293 were significantly higher than those of control. Expression of 1Ax1 also reduced the weakening slope. Unlike *1Ax1*-expressing lines, mixing parameters from lines P-121 and P-149 revealed marked differences in dough behaviors between *1Ax1*-expressing and control lines. The expression of PINA in lines P-121 and P-149 was associated with decreased curve widths with respect to non-transformed control, with MLW, MPW and MRW being significantly reduced. MPT was significantly higher for lines P-121 and P-149 than that for the control. But weakening slope was lower for these lines with respect to Luna. No significant difference was observed in the MTxI from *Pina*-expressing and control lines.

Differences in mixing behaviors between lines expressing *1Ax1* and/or *Pina* revealed the combined effects of 1Ax1 and PINA on dough properties. As shown in Figure 5, both the heights and widths of the curve were significantly higher for lines HP-19 and HP-245 as compared with those for lines expressing either *1Ax1* or *Pina*, with the exception of a non-significant increase of MRW observed in lines HP-19 and HP-245. Moreover, MPT was significantly increased for lines HP-19 and HP-245 with respect to the Luna control, but was similar to those for the *Pina*-expressing lines (P-121 and P-149). Non-significant reduction in WS was seen in lines HP-19 and HP-245 compared with that of Luna, the decreased WSs in HP-19 and HP-245, however, were similar to those in lines P-121 and P-149. These results demonstrated that lines expressing the combination of *1Ax1* and *Pina* did not differ in MPT and WS in comparison with lines expressing only *Pina*. More importantly, the MTxI for lines HP-19 and HP-245 were significantly increased compared with all the other transgenic lines and the Luna control, suggesting stronger doughs from lines HP-19 and HP-245 than those from the other lines.

## Discussion

Dough mixing is a critical process in the production of flour-based food and greatly influences the end-use quality of wheat that mainly derives from two traits: grain hardness and gluten protein [2]. On one hand, extensive studies have demonstrated that the HMW-GS plays a determinant role in dough properties and breadmaking quality of wheat [34]. In wheat, small-scale and large-scale rheological tests of transgenic wheat with expression of 1Ax1, 1Dx5 or 1Dy10 have confirmed that these three subunits can improve dough strength and are associated with good breadmaking quality [3], [35], [36], [37]. On the other hand, a few studies showed that grain hardness primarily controlled by *Pina* and *Pinb* genes had influences on milling and baking qualities [38], [39]. In addition, overexpression of *Pina* and *Pinb* in transgenic lines of bread wheat negatively affected crumb grain score and loaf volume [40]. Therefore, both *HMW-GS* and *Pin* genes should be taken into consideration in developing breeding strategy for improvement of wheat end-use quality. In this study, transgenic lines of durum wheat expressing *1Ax1* and/or *Pina* were screened out and allowed the separate and combined effects of 1Ax1 and PINA on dough mixing properties to be determined. A null segregant line (line N-2) and the non-transformed cv. Luna were used as controls. Both control lines were null background for the target genes (*1Ax1* and *Pina*) and marker genes (*nptII*, *uidA* and *bar*), whereas marker genes were present in transgenic lines expressing *1Ax1* and/or *Pina* (Figure S1). Previous studies on transgenic lines expressing HMW-GS genes have demonstrated that no differences were found in agronomic performance and rheological properties between lines constitutively expressing the marker genes from those which only expressed the HMW-GS genes [37], [41]. Furthermore, numerous studies supported that the use of selectable marker genes (nptII, uidA and bar) in transformation posed no safety concerns [42], and few studies showed these marker genes were related to phenotypic variations in transgenic lines of wheat. Therefore, it is very unlikely that the presence or expression of *bar*, *uidA* and *nptII* genes has effects on the grain hardness, dough properties, as well as agronomic performance of transgenic lines.

### Overexpression of PINA in Durum Wheat Leads to Medium Hard Grain Texture

Previous data indicated that friabilin is as a marker protein for grain hardness as it is abundant on the water-washed starch granules from soft wheat and scarce on hard wheat starch [12]. Then friabilin was found to be composed of PINA and PINB proteins which are encoded by two closely linked *Pin* genes at the *Ha* locus [11], [13]. Furthermore, the results that complementation of the mutant *Pina-D1b* or *Pinb-D1b* allele with their wild-type alleles restored a soft-texture phenotype demonstrate that *Pina* and *Pinb* genes are the major causal genes for grain hardness [16], [17], [18]. In addition, transgenic plants with expression of *Pina* and/or *Pinb* in rice and maize which are null background for the *Ha* locus had decreased grain hardness and lowered levels of damaged starch [15], [19]. However, unlike transgenic lines of wheat, transgenic rice and maize did not show apparent difference in grain hardness as large as those observed in wheat, indicating that the mechanisms for grain hardness in rice and maize may be different from that in wheat. In our work, overexpression of *Pina* in durum wheat resulted in similar phenotype (medium hard texture of grain) as observed in bread wheat [17]. Therefore, the results of transgenic lines presented here, together with results from durum wheat cv. Langdon and its substitution line Langdon 5D [15] prove that grain hardness can be modulated by PINA and PINB proteins in both durum wheat and bread wheat in a similar way. Previous data implicate that PINA and PINB proteins may determine grain hardness of wheat in some type of synergism. Expression of the wild-type *Pina-D1a* allele in a background with mutant *Pinb* led to medium-hard phenotype of grain texture [17]. Moreover, decrease in grain hardness for the addition of wild-type *Pina* (the *Pina-D1a* allele) to a *Pina*-mutant background was not as dramatic as the addition of wild-type *Pinb* (the *Pinb-D1a* allele) to a *Pinb*-mutant background [43], [44]. The *Pina*-expressing lines herein showed increased total and starch-bound PINA, as well as decreased grain hardness (Table 1 and Figure 2). Interestingly, regardless of larger variations in total PINA levels among *Pina*-expressing lines, similar levels of starch-bound PINA were observed these lines which are null background for functional PINB protein (Figure 2) indicates that PINB may be a limiting factor in PINA binding to starch and in reduction of grain hardness.

### Expression of Transgenic 1Ax1 is Compensated by other Storage Proteins

Expression patterns of endogenous storage proteins in transgenic lines of durum and bread wheat with overexpression of HMW-GS subunits have been extensively investigated in previous studies. In a field-grown transgenic line overexpressing *1Dx5*, expression levels of most endogenous HMW-GS subunits uniformly dropped about 30%, with an increase in the overall LMW-GS level and a decrease in gliadin level being observed [36]. Another group reported that increases in the transgenic 1Dx5 and/or 1Dy10 expression were compensated by the decrease in HMW-GS Ax-, Bx- and By- subunits and dropped LMW-GS levels were also detected [45]. Moreover, besides significant decreases in endogenous HWM-GS subunits reported in most transgenic events overexpressing HMW-GS, nonsignificant decreases in LMW-GS were also reported in recent studies on transgenic lines expressing *1Ax1*, *1Dx5* or *1Dy10* under field conditions [3], [37]. In the present work, we found that expression of transgenic 1Ax1 was compensated by the significant decrease in endogenous Bx- and By- subunits, with the overall LMW-GS levels being non-significant lower (Figure 3). Our results along with previous data indicate that additional expression of transgenic HMW-GS is strongly related to the decrease in endogenous HMW-GS and tend to reduce the overall LMW-GS expression levels. However, mechanisms underlying this compensation effects is largely unknown. One possibility is that the compensatory phenomenon may reflect the competition for amino acids for protein synthesis [46]. This hypothesis is supported by the “*in silico*” amino acid composition analysis of gluten proteins for transgenic wheat lines with down-regulation of γ-gliadins [47]. On the other hand, there are results against this hypothesis in rice and barley, where the available sulfur-containing amino acids may be key regulator in controlling the amino acid homeostasis [48], [49]. Nevertheless, the mechanism for storage protein compensation in wheat is worthy of in-depth investigation in future.

### Effects of 1Ax1 and/or PINA on Dough Mixing Properties

The dough mixing characteristics of transgenic and control lines were shown in Table 1. Relationships between Mixograph parameters and dough visco-elasticity have been explained in details [31] and associations of these parameters with other wheat quality traits were discussed previously [50]. Generally, MPW and MRW are positively correlated with dough resistance to extension [51], whereas WS and MTxW are, respectively, negatively and positively correlated with the over-mixing tolerance [31], [47]. MPV and MTxI are positively correlated with dough strength [3]. Weak dough has higher WS, shorter MPT and lower MPV and MTxI than those of strong dough. In our study, no differences were found in mixing properties between the null segregant line (N-1) and non-transformed control (Luna) was consistent with the identical protein patterns storage between these two control lines (Figure S2 and Figure 3), demonstrating that possible somaclonal variations in the transformation did not led to significant variations in storage proteins.

In transgenic lines exclusively expressing *1Ax1* (lines H-182 and H-293), significant increase of all parameters relating to the curve heights with respect to Luna demonstrated that dough elasticity was increased by expression of 1Ax1. Furthermore, increased MPT and MTxI for lines H-182 and H-293 indicated the enhanced dough strength in comparison with those for Luna. Lower WS values and non-significantly higher MTxW values for lines H-182 and H-293 revealed that the mixing tolerance was also improved by 1Ax1 expression. In previous studies, although subunit 1Ax1 was generally reported to have positive effects on dough strength in bread wheat, durum wheat and Tritordeum [5], [6], [7], [8], differential effects of 1Ax1 on dough functional properties were also revealed by rheological tests in transgenic lines with different wheat varietal backgrounds. In transgenic lines of cv. Bobwhite and Anza, both dough strength and mixing tolerance were increased by expression of transgenic 1Ax1 [3], [52]. Overexpression of 1Ax1 improved both dough strength and extensibility in transgenic lines of L88-31 [35]. Interestingly, a study on a series of transgenic lines of three commercial wheat cultivars (Imp, Canon and Cadenza) with expression of 1Ax1 showed that high expression levels of 1Ax1 conferred “over-strong” dough properties and had negative effects on breadmaking performance; whereas lower expression levels of 1Ax1 increased dough strength and stability [37]. In the cases of our study, expression of 1Ax1 led to improved dough strength and mixing tolerance, and decreased dough resistance to extension. Regarding the effects of 1Ax1 reported in this work as well as those reported previously, it should be born in mind that the effects of transgenic 1Ax1 on dough functionality may vary depending on the expression level of transgene and on the composition of endogenous HMW-GS, although usually overexpression of 1Ax1 improve dough strength.

In transgenic lines expressing only *Pina*, significant decreased curve-width-related mixing parameters (MPW and MRW) demonstrated that dough resistance to extension was reduced by expression of PINA. Moreover, slight but significant decrease of MPV and non-significant decrease of MTxI in *Pina*-expressing lines indicated that dough strength was slightly decreased by PINA expression. In addition, lower WS values for linesP-121 and P-149 with respect to non-transformed control indicated increased mixing tolerance. Previous data in transgenic lines of bread wheat expressing *Pina* and/or *Pinb* showed that transgenic addition of PINs detrimentally affected loaf volume and crumb grain score [40]. In soft-textured lines of durum wheat developed by chromosome engineering [53], Alveograph analysis revealed that reduction in grain hardness caused by the presence of the *Ha* locus, was related with drastic decrease in dough resistance to extension, and increased dough extensibility and over-mixing tolerance, with dough strength not significantly affected [4]. Partly agreed with Gazza et al. [4], our results confirmed the detrimental effect of PINA on dough resistance to extension and minor effect on dough strength. It is noteworthy that PINA and PINB interact with each other *in vivo* [54] and, *in planta*, control grain hardness in some type of synergism [43], [44]. Unlike the data reported by Gazza et al. [4], the results herein revealed the individual effects of transgenic PINA on dough mixing properties in a null background of durum wheat without any interaction with PINB.

In contrast with lines expressing either *1Ax1* or *Pina*, transformation with the combination of *1Ax1* and *Pina-D1a* resulted in better dough mixing properties. Significantly higher values of both curve-height-related mixing parameters (MLV, MPV, MRV and MTxV) and curve-width-related parameters (MLW, MPW, MTxW) from lines HP-19 and HP-245 than those from lines expressing either *1Ax1* or *Pina* demonstrated that coexpression of 1Ax1 and PINA improved dough strength and resistance to extension. Negative effects of PINA on dough properties were not found as detected in the *Pina*-expressing lines (P-121 and P-149); however, combined enhancing effects of 1Ax1 and PINA on dough strength resistance to extension were observed. The significantly higher MTxI for lines HP-19 and HP-245 with respect to lines expressing *1Ax1* or *Pina* further supports an enhancement of coexpressed 1Ax1 and PINA on dough strength. No significant difference was observed in WS among lines expressing *1Ax1* and/or *Pina*, suggesting that the over-mixing tolerance was not affected by the coexpressiong of 1Ax1 and PINA.

Interestingly, the finding of dough properties from these lines raises the question of why there were combined effects of 1Ax1 and PINA on dough mixing properties and how could coexpression of 1Ax1 and PINA lead to stronger dough than those from lines expressing either *1Ax1* or *Pina*. A remarkable difference between Mixograms from lines coexpressing *1Ax1* and *Pina* and those expressing only *1Ax1* is the shape of curves in the first two minutes of dough formation (Fig. 4). The beginning stages of dough development are the hydration stage and the blending stage. As described by Stauffer [33], protein network is gradually softened by water penetration in the hydration stage, with damage starch granules being absorbing water. As the protein network is agitated by mixing, all the ingredients of dough are being blended into a homogenous dough mass, with starch granules being less firmly associated with the protein fibers and lipids being uniformly distributed in the protein network. It has been documented that the hydration of dough formation depends on dough water absorption and starch damage level [55], [56]. Quantitative trait locus (QTL) identification analysis revealed that the *Ha* locus on chromosome 5D, consisting of *Pina* and *Pinb* genes, is a QTL for hydration traits including dough water absorption and damaged starch [57]. Further, it was demonstrated that expression of PINA and/or PINB led to enhanced grain softness and reduced starch damage levels in both transgenic rice and wheat [15], [16]. More recently, *Pin* genes’ association with damaged starch and water absorption were confirmed by Farinograph in soft-textured F_8_ lines of durum wheat containing the complete *Ha* locus [4]. Study of the hydration of wheat dough by tandem use of rheological tests and nuclear magnetic resonance (NMR) spectroscopy also supports the correlation between grain hardness and water absorption, finding that hard wheat required a higher water level to achieve a dough of satisfactory consistency [58]. Moreover, it was reported that PINA prevent absorption of lipids to air-water interfaces in dough and make lipids embedded within the protein-starch matrix, leading to a homogeneous size distribution of gas cells in wheat dough [59]. All the above leads up to an explanation for the difference in Mixogram shapes that decreased grain hardness caused by PINA expression could lead to lower levels of water absorption and damaged starch and hence result in faster hydration during mixing in comparison with those lines without transgenic *Pina*. Further, distribution of lipids in protein-starch matrix and lower levels of damaged starch in lines coexpressing *1Ax1* and *Pina* may help to form stronger dough than lines expressing *1Ax1*. This inference matches the higher values of MPV, MRV, MTxV and MTxI for lines HP-19 and HP-245 with respect to H-182 and H-293 (Figure 5). In addition, expressiong of *1Ax1* appears to compensate the detrimental effect of PINA on dough resistance to extension for unknown reason. In particular, due to the differential effects of transgenic 1Ax1 on dough properties in different genetic backgrounds as is discussed above [35], [37], [52], we can reasonably infer that the enhancing effects of 1Ax1 and PINA on dough mixing properties may possibly vary in durum wheat varieties with different HMW-GS compositions. Possible changes in the combined effects of 1Ax1 and PINA on dough functionality in different varietal backgrounds may be related with complicated interactions between starch and protein matrix during dough development and need intensive studies in future.

In summary, we have demonstrated that coexpression of 1Ax1 and PINA in durum wheat have combined effects on dough mixing behaviors with a better dough strength and resistance to extension than those from lines expressing *1Ax1* or *Pina*. Moreover, detrimental effect of PINA on dough resistance to extension was observed in *Pina-*expressing lines with respect to non-transformed cv. Luna, whereas coexpression of 1Ax1 and PINA improved dough strength and resistance to extension in comparison with the *1Ax1*-expressing lines. The results in our study showed that expression of HMW-GS could offset detrimental effects of PINA on dough mixing properties in durum wheat, and implicate that combinations of transgenic HMW-GS and PINs may lead to different dough properties of durum wheat, thus varied end-use qualities. Therefore, these results suggest that simultaneous modification of dough strength and grain hardness in durum wheat could significantly improve its breadmaking quality and may even not weaken its pastamaking potential. Furthermore, expression of PINs in durum wheat would likely decrease the energy consumption during milling due to the close relationship between grain hardness and milling energy [60]. For all these reasons, modification of both dough strength and grain hardness in durum wheat has practical implications for breeding durum wheat with dual functionality (for pasta and bread) and therefore may improve the economic values of durum wheat.

## Supporting Information

Figure S1 **PCR detection for the** ***nptII*** **,** ***bar*** **and** ***uidA*** **genes in transgenic and control lines.** The presence or absence of *nptII*, *bar* and *uidA* genes in genomic DNA were determined by PCR lines HP-19 (lane 1), HP-245 (lane 2), H-182 (lane 3), H-293 (lane 4), P-121 (lane 5), P-149 (lane 6), N-1 (lane 7) and non-transformed cv. Luna (lane 8). Lane 9 and lane 10 represented the plasmid control and water negative control of PCR amplification.
(TIF)

Figure S2 **No significant differences were found in dough mixing parameters between lines N-1 and Luna.** Dough mixing parameters for null segregant line (N-1, indicated by grey bars) and non-transformed control cv. Luna (WT, indicated by white bars) were compared by Student’s *t* test. All the eleven mixing parameters for line N-1 used in this study were not significant different from those for the Luna control.
(TIF)

Table S1 **Primers designed for PCR amplification of** ***bar*** **,** ***uidA*** **and** ***npt II*** **genes.**
(DOC)
