# Supplementary figures and images for: Coexpression of the High Molecular Weight Glutenin Subunit 1Ax1 and Puroindoline Improves Dough Mixing Properties in Durum Wheat (Triticum turgidum L. ssp. durum)
Source: PLoS One. 2012 Nov 21;7(11):e50057. doi: 10.1371/journal.pone.0050057 (PMC3503773; doi:10.1371/journal.pone.0050057)

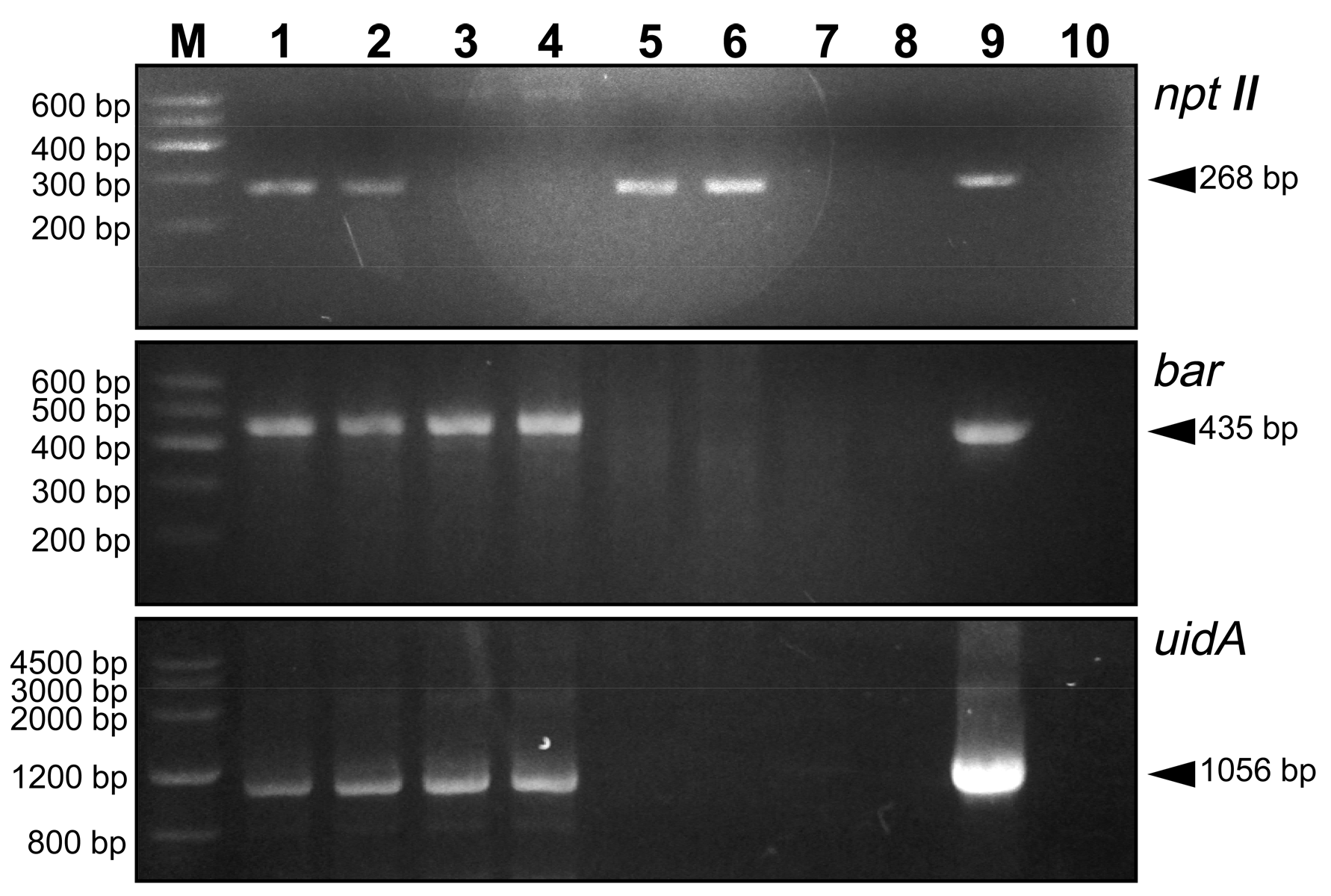

Supplement: Figure S1 — PCR detection for the nptII , bar and uidA genes in transgenic and control lines. The presence or absence of nptII, bar and uidA genes in genomic DNA were determined by PCR lines HP-19 (lane 1), HP-245 (lane 2), H-182 (lane 3), H-293 (lane 4), P-121 (lane 5), P-149 (lane 6), N-1 (lane 7) and non-transformed cv. Luna (lane 8). Lane 9 and lane 10 represented the plasmid control and water negative control of PCR amplification. (TIF) [file pone.0050057.s001.tif]

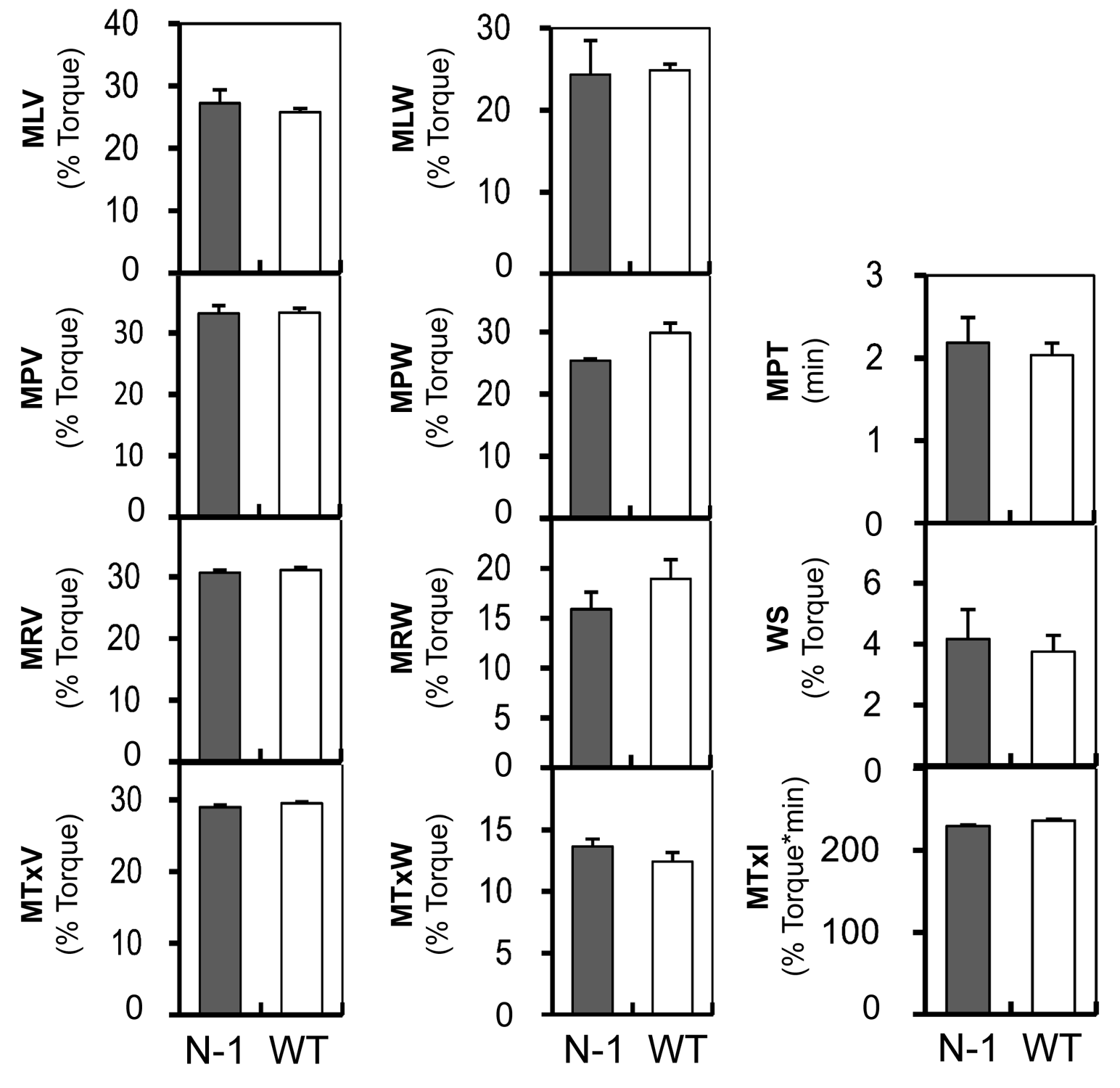

Supplement: Figure S2 — No significant differences were found in dough mixing parameters between lines N-1 and Luna. Dough mixing parameters for null segregant line (N-1, indicated by grey bars) and non-transformed control cv. Luna (WT, indicated by white bars) were compared by Student’s t test. All the eleven mixing parameters for line N-1 used in this study were not significant different from those for the Luna control. (TIF) [file pone.0050057.s002.tif]
