# Supplementary material for: Coexpression of the High Molecular Weight Glutenin Subunit 1Ax1 and Puroindoline Improves Dough Mixing Properties in Durum Wheat (Triticum turgidum L. ssp. durum)
Source: PLoS One. 2012 Nov 21;7(11):e50057. doi: 10.1371/journal.pone.0050057 (PMC3503773; doi:10.1371/journal.pone.0050057)
Supplement: Table S1 — Primers designed for PCR amplification of bar , uidA and npt II genes. (DOC) [file pone.0050057.s003.doc]

Supplemental Information

**Table S1. Primers designed for PCR amplification of *bar*, *uidA* and *nptⅡ* genes.**

| Primer name | Sequences of primers (5'–3') | Amplification product size (bp) | Anneal temperature (℃) | Target gene |
| --- | --- | --- | --- | --- |
| npt-F | GCTATGACTGGGCACAACA | 268 | 58 | *nptⅡ* |
| npt-R | GCAGGAGCAAGGTGAGATG | 268 | 58 |  |
| bar-F | CTGCACCATCGTCAACCACTACATC | 435 | 60 | *bar* |
| bar-R | AGCTGCCAGAAACCCACGTCAT | 435 | 60 |  |
| uidA-F | agtgtacgtatcaccgtttgtgtgaac | 1056 | 62.5 | *uidA* |
| uidA-R | atcgccgctttggacataccatccgta | 1056 | 62.5 |  |
